# Performance status is the most powerful risk factor for early death among patients with advanced soft tissue sarcoma The European Organisation for Research and Treatment of Cancer – Soft Tissue and Bone Sarcoma Group (STBSG) and French Sarcoma Group (FSG) study

**DOI:** 10.1038/bjc.2011.136

**Published:** 2011-04-19

**Authors:** N Penel, M V Glabbeke, S Mathoulin-Pelissier, I Judson, S Sleijfer, B Bui, P Schoffski, M Ouali, S Marreaud, V Brouste, A Duhamel, P Hohenberger, J-Y Blay

**Affiliations:** 1 Department of General Oncology, Centre Oscar Lambret, Regional Comprehensive Cancer Centre, 3, rue F Combemale, 59020, Lille, France; 2 European Organisation for Research and Treatment of Cancer, Brussels, Belgium; 3 Department of Clinical Epidemiology and Clinical Research, Institut Bergonié, Regional Comprehensive Cancer Centre, Bordeaux, France; 4 Sarcoma Unit, Royal Marsden Hospital, London, UK; 5 Department of Medical Oncology, Erasmus Cancer Center, Rotterdam, The Netherlands; 6 Department of Medical Oncology, Institut Bergonié, Regional Comprehensive Cancer Centre, Bordeaux, France; 7 Department of General Medical Oncology, Leuven Cancer Center, Leuven, Belgium; 8 Public Health Unit (EA 2694: Epidemiology and Modelization of Chronic Illness), Lille University, Lille, France; 9 Division of Surgical Oncology and Thoracic Surgery, Department of Surgery, Medical Faculty Mannheim, University of Heidelberg, Mannheim, Germany; 10 Léon Bérard Comprehensive Cancer Centre, University Claude Bernard Lyon I, Lyon, France

**Keywords:** soft tissue sarcoma, early death, prediction

## Abstract

**Background:**

We investigated prognostic factors (PFs) for 90-day mortality in a large cohort of advanced/metastatic soft tissue sarcoma (STS) patients treated with first-line chemotherapy.

**Methods:**

The PFs were identified by both logistic regression analysis and probability tree analysis in patients captured in the Soft Tissue and Bone Sarcoma Group (STBSG) database (3002 patients). Scores derived from the logistic regression analysis and algorithms derived from probability tree analysis were subsequently validated in an independent study cohort from the French Sarcoma Group (FSG) database (404 patients).

**Results:**

The 90-day mortality rate was 8.6 and 4.5% in both cohorts. The logistic regression analysis retained performance status (PS; odds ratio (OR)=3.83 if PS=1, OR=12.00 if PS ⩾2), presence of liver metastasis (OR=2.37) and rare site metastasis (OR=2.00) as PFs for early death. The CHAID analysis retained PS as a major discriminator followed by histological grade (only for patients with PS ⩾2). In both models, PS was the most powerful PF for 90-day mortality.

**Conclusion:**

Performance status has to be taken into account in the design of further clinical trials and is one of the most important parameters to guide patient management. For those patients with poor PS, expected benefits from therapy should be weighed up carefully against the anticipated toxicities.

Soft tissue sarcomas (STSs) account for approximately 1 to 2% of all adult cancers. Although local control can be obtained through the use of surgery plus radiotherapy, up to 50% of patients will recur at distant sites (Clark *et al*, 2005). At the metastatic stage, palliative chemotherapy can be considered as a reasonable option in the majority of cases. Both ifosfamide and doxorubicin are the best single agents with activity in the treatment of STS. In general, the toxicities are manageable but real, thereby making treatment difficult (Sleijfer *et al*, 2005; Benjamin 1987). However, most if not all patients will ultimately relapse and die of their disease. The median overall survival (OS) is actually ∼12 months and the median time to progression is ∼3 months. Regarding these facts, three options could be considered for the treatment of patients with advanced/metastatic STS: (1) the combination of doxorubicin and ifosfamide if resection of metastasis looks feasible or in the case of symptomatic patients with rapidly progressing tumours (2) single-agent chemotherapy and (3) exclusive best supportive care (Blay *et al*, 2003; Benjamin 1987). In the everyday practice, many factors are integrated in the decision-making process: general condition of patient, underlying co-morbidities, medical history, resectability of metastasis, patient choice and knowledge of prognostic factors (PFs).

One of the most important factors that should be taken into account is the risk that a patient will die early after initiation of treatment. Particularly in patients with a high risk of dying early, it is likely that best supportive care should be preferred over systemic therapy with its accompanying toxicities.

In addition to patient care, insight into factors associated with early death after initiation of treatment is crucial for the design of studies, as the lack of reliable guidance for the life expectancy prediction is likely to introduce some biases in these clinical studies. Moreover, the known PFs for outcome have to be taken into account in the design of clinical trials, for example, as stratification factors at entry for randomised clinical trials (Simon and Altman, 1994).

The literature shows that life expectancy is most often overestimated, which frequently results in overtreatment (Maltoni *et al*, 1994; Christakis and Lemont, 2000; Penel *et al*, 2008; Clément-Duchêne *et al*, 2010). For example, despite the fact that a life expectancy of <3 months is an exclusion criterion for all phase I studies, without reliable guidance, ∼20–30% of patients enroled in phase 1 trials in expert centres died within the first 90 days (Arkenau *et al*, 2008, 2009; Penel *et al*, 2008). Despite the high need for models establishing the risk for early death and a large body of literature on prognostic models for cancer patients, only few of these models are suitable for daily decision making. Before implementation in clinical practice, such prognostic models need to be identified and subsequently validated in independent series. Additionally, such models should be robust and simple, in order to be easily applied in daily practice.

Regarding the severity of advanced STS and the limited therapeutic options, we carried out a retrospective exploratory analysis to (1) develop prognostic models for early death in such population and (2) then validate the models in an independent data set. For the purpose of this study, two complementary approaches were used for the development of the models (scoring system derived from logistic regression analysis and algorithm derived from decision-tree analysis). The models were developed with the Soft Tissue and Bone Sarcoma Group (STBSG) data set and subsequently validated with the French Sarcoma Group (FSG) data set.

## Materials and methods

### Data sets

We used two data sets. The first one was formed by the STBSG and includes patients treated with single agents and combination regimens as first-line treatments between January 1976 and October 2001. The second one had been built by the FSG and includes patients treated with combination regimen (MAID and intensified-MAID) between January 1994 and October 2008 (Fayette *et al*, 2009; Bui *et al*, 2009).

### Primary end point

The primary end point was 90-day mortality (early death). This threshold is believed to be relevant in decision making for advanced cancer patients in whom the choice of whether to treat with chemotherapy or best supportive care need to be discussed (Sessa *et al*, 1996; Geraci *et al*, 2006; Kelly *et al*, 2007; Penel *et al*, 2009).

### Development of the models

The development of these models used the STBSG database. The potential PFs were: gender, age, performance status (WHO-PS), histological subtypes (angiosarcoma, fibrosarcoma, leiomyosarcoma, malignant histiocytofibroma, malignant peripheral sheath nerve tumour, rhabdomyosarcoma, synovial sarcoma, unclassified STS and unknown subtype and a group containing the remaining rare subtypes), histological grade, tumour location (abdominal, breast, gynaecological, head and neck, lower limbs, skin, trunk upper limbs, visceral, other locations and unknown), time interval since initial diagnosis of STS and start of systemic therapy, presence of lung metastasis, presence of liver metastasis, presence of bone metastasis and presence of other metastases. Two continuous variables (age and time interval since initial diagnosis of STS) were recorded according to the observed quartiles. All these items were collected in both databases.

Two complementary approaches of logistic regression analysis and classification analysis (CHAID) (Melchior *et al*, 2001; Barton *et al*, 2005; Chan *et al*, 2006; Ambalavanan *et al*, 2006; Courville *et al*, 2009) were used for development of the prognostic models (scoring system and decision tree, respectively).

Separate logistic regression analysis with stepwise selection of variables (at *P*<0.05) identified variables in the entire STBSG cohort associated with early death. The odds ratios (ORs) from the logistic regression model were converted into points by dividing by the smallest OR for any given other OR. Next, an overall score was assigned to each patient by summing the points they received for each of the PFs.

In the current study, CHAID was used as a complementary method; this technique uses a systematic algorithm to detect the stronger association between potential PFs (named ‘splitter’) and the outcome variable (e.g., early death). Step by step, the CHAID algorithm recursively partitions data into mutually exclusive, exhaustive subsets that are maximally different in the dependent variable (e.g., early death), as assessed with the use of Bonferroni-adjusted *χ*^2^ statistics. The CHAID algorithm consisted, herein, in three successive actions: (1) merge the subgroups with similar occurrence of target variable (*α*_merge_=0.01), (2) split the subgroups using the best PF (*α*_splitt_=0.01) and (3) terminate the tree when the observed number of early death was ∼30 (Melchior *et al*, 2001; Barton *et al*, 2005; Chan *et al*, 2006; Ambalavanan *et al*, 2006; Courville *et al*, 2009).

Furthermore, the prognostic accuracy (and its 95% confidence intervals (95% CIs)) was tabulated for both models (scoring system and decision-tree analysis) using a classical 2 × 2 table. The optimal threshold for each prognostic model was established using classical receiver operating characteristics analysis to maximise both sensitivity and specificity (Linden 2006). The accuracy is basically the rate of well-classified patients.

### Validation and head-to-head comparison of both models

Both models were applied to the independent data set (FSG database). The score was calculated for each patient according to the observed presence of PFs (a patient with any missing source variable was given a missing value of score). The entire population was split according to the proposed CHAID algorithm. Performance of both models had been established using area under receiver operator curve and accuracy as previously specified.

## Results

### Patient characteristics

The main patient characteristics are depicted in Table 1. The STBSG cohort included 3002 patients; 2898 (96.5%) of them were assessable for the early death rate. The rate of early death was 251 out of 2898 (8.6% (95% CI: 7.6–9.6)). In this cohort, the median OS was 348 days. Out of the 404 patients from the FSG cohort, 399 (98.7%) were assessable for the early death rate. The rate of early death was 18 out of 399 (4.5% (2.4–6.5)). The median OS of this cohort was 788 days.

### Development of the scoring system

The following parameters were associated with the risk of early death in univariate analysis: age ⩾60 (OR=1.77, *P*=0.003), PS=1 (OR=3.04, *P*<0.0001), PS ⩾2 (OR=10.00, *P*<0.0001), time interval since the initial diagnosis of sarcoma ⩾540 days (OR=0.41, *P*<0.0001), presence of liver metastasis (OR=2.37, *P*=0.0041) and presence of other metastases (OR=2.00, *P*=0.0061; Table 2). The final logistic regression analysis retained the following PFs for early deaths: PS=1 (*P*<0.0001), PS ⩾2 (*P*<0.0001), presence of liver (*P*=0.0014) or other metastases (*P*=0.0055). For the attribution of points in the scoring system, each adjusted OR was divided by the smallest one, which appeared to be the presence of metastases other than lung, bone and liver with an OR of 2.0. Thus, PS=1 then gave 2 points (3.83/2.00), PS ⩾2 gave 6 points (12.00/2.00), presence of liver metastasis gave 1 point (2.37/2.00) and presence of other metastases also gave 1 point (2.00/2.00). As a result, the sum of points ranged from 0 to 8 and the risk of early death from 3 to 40% (Table 3). The area under receiver operator curve was 0.69 (0.66–0.73). The optimal threshold of this scoring system was set at 3. Using this threshold, the prognostic accuracy was 91.0% (90.5–0.92.7), the positive predictive value was 22.0% (17.3–27.3) and the negative predictive value was 88.7% (87.4–89.4).

### Development of the decision tree

**Figure 1:**
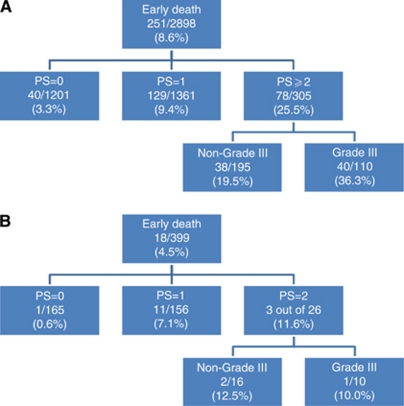
CHAID algorithms. (**A**) STBSG data set and (**B**) FSG data set.

The CHAID analysis provided a very simple algorithm. In the decision tree, the most powerful discriminator (splitter) was the PS; three subsets of patients were discriminated with increasing risk of early death: patients with PS=0 (early death rate: 3.3%), patients with PS=1 (early death rate: 9.4%) and patients with PS ⩾2 (early death rate: 25.5%). There was no discriminator able to split the two first categories of patients. In the development data set, among patients with PS ⩾2, the histological grade was able to individualise two subsets of patients; when the grade was 3, the rate of early was 36.3% and in the other situations, the rate of early death was 19.5% (Figure 1).The area under the receiver operator curve was 0.67 (0.64–0.71). The optimal classification was based on the separation of patients with PS=(0–1) from other patients (Table 4). Using this classification, the prognostic accuracy was 86.2% (84.5–87.4), the positive predictive value was 25.3% (20.4–30.1) and the negative predictive value was 93.3% (92.0–94.6).

### Validation and head-to-head comparison of both models

Both models were applied to the FSG cohort. The scoring system was applicable to 249 out of 404 patients (61.6%). In this cohort, the area under receiver operator curve was 0.68 (0.52–0.83). In this validation cohort, the prognostic accuracy of this scoring system, with a threshold set at 3, was 67.7%. The CHAID algorithm was applicable to 347 out of 404 patients (85.9%). Among patients with PS ⩾2, the rates of early death were similar whatever the histological grade. In this cohort, the area under receiver operator curve was 0.72 (0.58–0.86). In this validation cohort, the prognostic accuracy of the CHAID algorithm was 89.0% (86.5–93.5). In the development cohort, the prognostic accuracy of the CHAID algorithm was superior to the one of the scoring.

**Figure 2:**
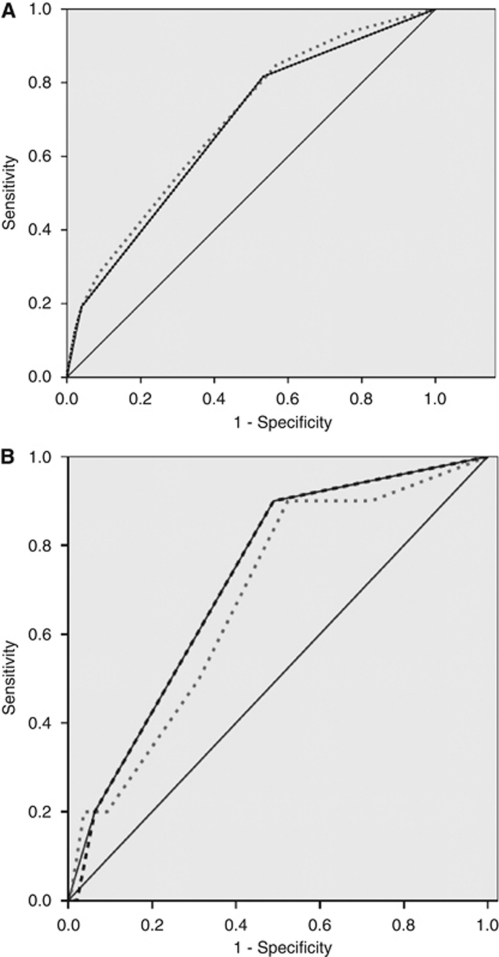
Area under receiver operator curves. (**A**) STBSG database. Grey dotted line: logistic regression-based scoring; black dotted line: *χ*^2^ interaction detection algorithm; black line: performance status alone as prognostic model (this line completely covers the CHAID algorithm line). (**B**) FSG database. Grey dotted line: logistic regression-based scoring; black dotted line: *χ*^2^ interaction detection algorithm; black line: performance status alone as prognostic model (this line completely covers the CHAID algorithm line).

Because PS appears as the most important PF in both models, we evaluated the accuracy of PS as a PF for early death (see Figure 2). In the validation cohort, the area under the receiver operator curve of PS was similar to both other models: 0.72 (0.58–0.86).

## Discussion

In both cohorts consisting of patients who were treated in the context of clinical trials with clear predefined eligibility criteria, the rates of early death were relatively low (8.6 and 4.5%). The rate of early death was significantly lower in the most recent trials (the French Sarcoma Group ones) that explored the role of poly-chemotherapy (MAID regimen) and the role of intensive chemotherapy. Hence, it is not surprising that the rate of early death was lower in trials with more stringent eligibility criteria. We have observed that the rate of early death in this population is lower than the reported one among patients entering in phase 1 trials (∼16%) or in patients with carcinoma of unknown primary (30%) (Geraci *et al*, 2006; Kelly *et al*, 2007; Arkenau *et al*, 2008; Penel *et al*, 2008, 2009; Ferté *et al*, 2010). The present analysis shows that PS is the most powerful PF for early death among patients with advanced STS treated with first-line systemic therapy. Using two complementary approaches, we developed and validated two prognostic models, which were however essentially based on the assessment of PS. The scoring system was more complex, incorporating three parameters, and was therefore available in a more limited part of the study population. The CHAID algorithm was based on twp variables (PS and histological grade) and was available in a larger proportion of patients. The implementation of the CHAID algorithm to the validation data set had showed that the second splitter (histological grade) did not improve the discrimination obtained with the first splitter (PS). Lastly, the prognostic performance of PS alone was as good as both other more sophisticated models (Figure 2).

The list of factors associated with early death identified in the present study is not surprising, and most of the identified factors have previously been revealed in other advanced STS databases as being related to outcome. The impact of presence of extra-pulmonary and especially liver metastasis had been mentioned as a PF for OS in non-pretreated patients (Karavasilis *et al*, 2008; Ray-Coquard *et al*, 2009). Grade has also been identified as a PF for OS in non-pretreated patients (Antman *et al*, 1993). The time interval since the initial diagnosis of STS has been identified as a PF for OS in non-pretreated patients by Maurel *et al* (2009). Performance status appeared as a PF for OS in most studies (Borden *et al*, 1987; Karavasilis *et al*, 2008; Maurel *et al*, 2009; Ray-Coquard *et al*, 2009). The database used for the development of these predictive models for early death had previously been used for analysis of other PFs. Van Glabbeke *et al* (1999) found that the following factors were associated with OS in patients who received doxorubicin: PS (HR=1.51, *P*<0.0001), liver metastasis (HR=1.46, *P*<0.0001), histological grade (HR=1.24, *P*=0.0002), time since initial diagnosis (HR=0.92, *P*=0.0004) and age (HR=1.10, *P*=0.0045). In the same study, the following parameters were associated with objective response: liver metastasis (OR=0.38, *P*<0.0001), age (OR=0.83, *P*=0.0024) and histological grade (OR=1.35, *P*=0.051). Blay *et al* (2003) identified the following parameters as factors associated with long-term survival: PS (OR=2.02, *P*=0.0235), grade (OR=2.18, *P*=0.0009), female (OR=0.43, *P*=0.0291) and complete response obtained after first-line treatment (OR=0.11, *P*=0.0001). The originality of this work is that the relative weight of each PF and its interaction were studied in a very large data set using two complementary approaches, and the identified factors were validated in an independent cohort. At the end, the prognostic value of PS outweighs all other clinical parameters.

The limitations of this study are related to its retrospective nature. For example, grade scoring and pathological classification change during the study period. The prognostic value of grade scoring is not established for some particular histological sub-types (such as clear cell sarcoma, epithelioid sarcoma, angiosarcoma and so on). Moreover, the parameters used herein are the basic clinical variables. For other tumour types, more elaborate and sophisticated scores have been published, which integrate other parameters such as lymphocyte count, LDH level or albumin (Geraci *et al*, 2006; Kelly *et al*, 2007; Arkenau *et al*, 2008; Penel *et al*, 2008, 2009). These parameters were not available in a sufficiently large section of the STBSG cohorts and could not be explored in the present study. The additional value of these biological parameters should be evaluated. Furthermore, one could argue that the development of prognostic model for early death might be more relevant in patients failing first-line chemotherapy and candidates for second-line treatment. We plan this second analysis. Moreover, we ignore the precise cause of the death in both databases, especially the incidence of toxic death *vs* death caused by progressive disease.

Despite its subjective nature, estimation of general condition by PS remains one of the most powerful PF in advanced/metastatic STS patients. Nevertheless, PS is not suitable for disabled and/or patients who suffer with persistent pain, for example, patients treated with previous mutilating surgical procedure. There are several scales available for scoring PS: the WHO-PS scale, the ECOG-PS scale or the Karnofsky PS scale. These different scales can generate discrepancies for evaluation of PS. It is well known that physicians overestimate both PS and life expectancy (Parkes, 1972; Evans and McCarthy, 1985; Christakis and Lemont, 2000; Ando *et al*, 2001). Ando *et al* (2001) had demonstrated that nurses and patients themselves estimate more accurately the actual PS than physicians.

Nevertheless, regarding its prognostic value, this variable has to be taken into account in future clinical trials, for example, as stratification factors at inclusion in randomised trials. For clinical decision making, both models developed are far from ideal. The probability of early death for a patient with PS ⩾2 is ∼11–24% compared with 0–3% for those with PS=0 (Figure 1). In everyday practice, this does not imply that all patients with PS ⩾2 should be denied palliative chemotherapy *per se*, but the high risk of an early death and the potential lack of benefit from treatment should be discussed with the patient before a decision is made to proceed with chemotherapy. Further studies are warranted to develop and validate more accurate prognostic models, if possible based on objectively measurable variables (such as biological parameters) and excluding PS.

## Figures and Tables

**Table 1:** The characteristics of patients in both cohorts

**Categorical variables**		**STBSG**	**FSG**
**Parameters**	**Categories**	**Cases (%)**	**Cases (%)**
Gender	Unknown	78 (2.6)	0 (0)
	Men	1464 (48.8)	192 (47.6)
	Women	1460 (48.6)	211 (52.4)
			
Performance status	Unknown	140 (4.7)	49 (12.2)
	0	1201 (40.0)	166 (41.2)
	1	1361(45.3)	157 (39.0)
	2	295 (9.8)	31 (7.7)
	3	5 (0.2)	0 (0)
			
Interval	Unknown	243 (8.1)	50 (12.3)
	0–60 days	841 (28.0)	274 (67.9)
	61–180 days	473 (15.8)	0 (0)
	181–540 days	723 (24.1)	0 (0)
	>540 days	722 (24.1)	80 (19.8)
			
Histological subtypes	Unknown	177 (5.9)	0 (0)
	Angiosarcoma	120 (4.0)	17 (4.2)
	Fibrosarcoma	178 (5.9)	8 (2.0)
	Leiomyosarcoma	907 (30.2)	108 (26.8)
	Liposarcoma	260 (8.7)	44 (10.9)
	MHF	303 (10.1)	20 (5.0)
	MPNST	144 (4.8)	16 (4.0)
	Other	292 (9.7)	68 (16.9)
	Rhabdomyosarcoma	104 (3.5)	22 (5.5)
	Synovial sarcoma	249 (8.3)	34 (8.4)
	Unclassified	268 (8.9)	66 (16.4)
			
Grade	Unknown	1122 (37.4)	25 (6.2)
	I	267 (8.9)	20 (5.0)
	II	707 (23.6)	107 (26.6)
	III	906 (30.2)	177 (43.0)
	Not applicable	0 (0)	74 (18.4)
			
Location	Unknown	1068 (35.6)	1 (0.2)
	Abdominal	409 (13.6)	67 (16.6)
	Breast	23 (0.8)	0 (0)
	Gynaecological	281 (9.4)	56 (13.9)
	Head and neck	78 (2.6)	18 (4.5)
	Lower limbs	507 (16.9)	138 (34.2)
	Other	18 (0.6)	8 (2.0)
	Skin	15 (0.5)	2 (0.5)
	Trunk	231 (7.7)	23 (6.9)
	Upper limbs	170 (5.7)	31 (7.7)
	Visceral	202 (6.7)	54 (13.4)
			
Lung met.	Unknown	134 (4.5)	125 (31.0)
	No	1280 (42.6)	86 (21.3)
	Yes	1588 (52.9)	192 (47.6)
			
Liver met.	Unknown	270 (9.0)	125 (31.0)
	No	2211 (73.7)	230 (57.1)
	Yes	521 (17.4)	48 (11.9)
			
Bone met.	Unknown	602 (20.1)	126 (31.3)
	No	2122 (70.7)	240 (59.6)
	Yes	278 (9.3)	37 (9.2)
			
Other met.	Unknown	639 (21.3)	126 (31.3)
	No	1329 (44.3)	240 (59.6)
	Yes	1034 (34.4)	37 (9.2)
			
Previous chemotherapy	Unknown	129 (4.3)	0 (0)
	No	2825 (94.1)	404 (100)
	Yes	48 (1.6)	0 (0)

Abbreviations: STBSG=Soft Tissue and Bone Sarcoma Group; FSG=French Sarcoma Group; MHF=malignant histiocytofibroma; MPNST=malignant peripheral nerve sheath tumour; met.=metastasis.

**Table 2:** Risk factors for early death (development data set)

	**Univariate analysis**			**Multivariate analysis**		
**Parameters**	**RR (e^*β*^)**	**95% CI**	***P*-value**	**RR (e^*β*^)**	**95% CI**	***P*-value**
*Age*						
40–49 *vs* <40	1.32	0.87–2.00	0.1816			
50–59 *vs* <40	1.41	0.95–2.08	0.8450			
⩾60 *vs* <40	1.77	1.21–4.61	0.0030			
Gender: female *vs* male	1.04	0.80–1.35	0.7586			
						
*Performance status*						
1 *vs* 0	3.04	2.11–4.36	<.0001	3.83	2.06–7.12	<.0001
2–3 *vs* 0	10.00	6.62–14.82	<.0001	12.00	5.53–26.06	<.0001
						
*Interval* ^a^						
Q2 *vs* Q1	0.80	0.55–1.17	0.2618			
Q3 *vs* Q1	0.87	0.63–1.20	0.4528			
Q4 *vs* Q1	0.41	0.27–0.61	<.0001			
						
*Histological subtype*						
MFH *vs* leiomyo	1.32	0.82–2.10	0.244			
Fibro *vs* leiomyo	0.59	0.21–1.68	0.325			
Lipo *vs* leiomyo	0.72	0.38–1.33	0.314			
Angio vs leiomyo	0.94	0.36–2.44	0.899			
Syno *vs* leiomyo	0.23	0.08–0.65	0.005			
MPNST *vs* leiomyo	0.48	0.21–1.07	0.074			
Rhabdo *vs* leiomyo	0.95	0.59–1.15	0.818			
Unclassified *vs* leiomyo	1.20	0.65–2.21	0.558			
Other *vs* leiomyo	0.91	0.40–2.06	0.826			
						
*Grade*						
II *vs*	1.39	0.77–2.95	0.2749			
III *v* I	1.99	1.13–3.49	0.0164			
						
*Primary site*						
Head/neck *vs* limb	1.88	0.88–4.00	0.1029			
Trunk *vs* limb	1.54	1.03–2.30	0.0338			
Viscera *vs* limb	1.61	1.06–2.45	0.0249			
Lung met.: yes *vs* no	1.07	0.83–1.40	0.5706			
Liver met.: yes *vs* no	1.57	1.19–2.14	0.0041	2.377	1.39–4.05	0.0014
Bone met.: yes *vs* no	1.13	0.73–1.74	0.5690			
Other met.: yes *vs* no	1.50	1.12–2.01	0.0061	2.002	1.22–3.27	0.0055

Abbreviations: CI=confidence interval; RR=relative risk; MHF=malignant histiocytofibroma; MPNST=malignant peripheral nerve sheath tumour; met.=metastasis.

a Q1: 0–60 days; Q2: 61–180 days; Q3: 181–540 days; and Q4:>540 days.

**Table 3:** Early death rates according to the score and score accuracy in both cohorts

	**STBSG data set**		**FSG data set**	
**Score**	***n***	**Early death (%)**	***n***	**Early death (%)**
0	472	11 (3)	68	1 (1.4)
1	408	13 (4)	46	0 (0)
2	564	46 (8)	53	4 (7.5)
3	508	51 (10)	54	3 (5.5)
4	110	15 (14)	7	0 (0)
6	74	18 (25)	7	0 (0)
7	100	26 (26)	13	3 (23)
8	26	10 (40)	1	0 (0)
Area under receiver operator curve	0.91 (0.90–0.93)		0.68 (0.62–0.73)	
		
	STBSG (*n*=2262)		FSG (*n*=249)	
	Early death	Other	Early death	Other
Score ⩾3	69	241	6	76
Score <3	121	1831	5	162
Accuracy (%) (95% CI)	91.0 (90.5–92.7)		67.7 (61.9–73.6)	

Abbreviations: STBSG=Soft Tissue and Bone Sarcoma Group; FSG=French Sarcoma Group; CI=confidence interval.

**Table 4:** Early death rate according to probability tree analysis and algorithm accuracy in both cohorts

**Groups**	**STBSG**		**FSG**	
PS=0	1201	40 (3.3)	165	1 (0.6)
PS=1	1361	129 (9.4)	156	11 (7.1)
PS ⩾2 and non-grade III	195	38 (13.9)	16	2 (12.5)
PS ⩾2 and grade III	110	40 (36.4)	10	1 (10.0)
Area under receiver operator curve	0.86 (0.84–0.87)		0.89 (0.86–0.93)	
		
	STBSG (*n*=2867)		FSG (*n*=347)	
	Early death	Other	Early death	Other
PS ⩾2	78	227	3	23
PS=(0–1)	169	2393	12	309
Accuracy (%) (95% CI)	86.2 (84.5–87.4)		89.0 (86.5–93.5)	

Abbreviations: STBSG=Soft Tissue and Bone Sarcoma Group; FSG=French Sarcoma Group; CI=confidence interval; PS=performance status.
